# Dermatophyte Infections Worldwide: Increase in Incidence and Associated Antifungal Resistance

**DOI:** 10.3390/life14010001

**Published:** 2023-12-19

**Authors:** Caroline Kruithoff, Ahmed Gamal, Thomas S. McCormick, Mahmoud A. Ghannoum

**Affiliations:** 1 Heritage College of Osteopathic Medicine, Ohio University, Cleveland, OH 44122, USA; ck784121@ohio.edu; 2 Center for Medical Mycology and Integrated Microbiome Core, Department of Dermatology, Case Western Reserve University, Cleveland, OH 44106, USA; axg1117@case.edu (A.G.); tsm4@case.edu (T.S.M.); 3 Department of Dermatology, University Hospitals Cleveland Medical Center, Cleveland, OH 44106, USA

**Keywords:** incidence, terbinafine, dermatophytes, trichophyton, *T. rubrum*, *T. mentagrophytes*, *T. indotineae*, superficial fungal infection

## Abstract

The increase in incidence of superficial fungal infections combined with the emergence of antifungal resistance represents both a global health challenge and a considerable economic burden. Recently, dermatophytes, the main culprit causing superficial fungal infections, have started to exhibit antifungal resistance. This can be observed in some of the most common species such as *Trichophyton rubrum* and *Trichophyton mentagrophytes*. Importantly, the new subspecies, known as *Trichophyton indotineae*, has been reported to show high resistance to terbinafine, a first-line treatment for dermatophyte infections. Compounding these issues is the realization that diagnosing the causative infectious agents requires using molecular analysis that goes beyond the conventional macroscopic and microscopic methods. These findings emphasize the importance of conducting antifungal susceptibility testing to select the appropriate antifungal necessary for successful treatment. Implementing these changes may improve clinical practices that combat resistant dermatophyte infections.

## 1. Introduction

Superficial fungal infections (SFIs) affect approximately 20 to 25 percent of the global population [1]. They can result in a myriad of dermatologic clinical presentations depending on both the organism involved and the area of the body affected [1]. Factors such as age, gender, and geographical location play an important role in the prevalence of these infections [2]. In a 2004 study in partnership with the American Academy of Dermatology, the prevalence of SFIs was reported to be 29.4 million cases. Between 1995 and 2004 there have been approximately 51 million patient visits for these infections [3,4]. Furthermore, between the years of 2005 and 2014, dermatophyte infections were responsible for 4,981,444 outpatient visits in the United States as reported by the Centers for Disease Control and Prevention (CDC). Moreover, the direct medical cost caused by these infections was approximately USD 845 million dollars in 2019. The worldwide increased incidence of fungal infections and growing trend of resistant organisms have attracted global concern [5,6]. While the exact reason behind this trend is under investigation, many factors have been reported as potential contributors, such as genetics, environmental factors, and antifungal resistance [5,6].

## 2. Epidemiology of Dermatophyte Infections

Dermatophytes are the most prevalent pathogenic fungi in the United States and amongst the most common causes of skin diseases worldwide [7]. Dermatophytes can be classified based on their habitat into anthrophillic (growing on humans), zoophilic (growing on animals), and geophilic (growing in soil) [8]. They belong to seven clades from A to G: clade A contains the *Trichophyton* species, clade B contains *Epidermophyton floccosum* species, and clade C & F contain the *Microsporum* species [9,10]. There are over 40 species of dermatophytes known to infect humans, primarily causing SFIs [11]. As a keratinophilic fungus (i.e., exhibiting affinity to keratin), dermatophytes infect the keratin structures of the skin, hair, and nails, resulting in an inflammatory host response and clinical conditions known as tinea [12]. These dermatophytes can also colonize human hosts without causing disease [11]. While the prevalence of dermatophyte species varies around different regions of the world, *Trichophyton rubrum* is responsible for the majority of dermatophyte-associated infections reported [11,12].

As dermatophytes thrive in hot, humid environments, many tropical and developing countries are facing an increase in dermatophyte infections [5]. Specifically, India has encountered an enormous challenge due to an alarming increase in the number of chronic and recurrent dermatophyte infections. The tropical and subtropical climate of the country is particularly favorable for dermatophytes [13]. Furthermore, overcrowding, shared living spaces, and urbanization are all contributing factors to the increasing prevalence of dermatophytosis [14]. In addition to the rapidly rising rate of infection, treatment efficacy has been sub-optimal due to a lack of antifungal stewardship in clinical practice [15]. Importantly, it is essential to consider non-dermatophyte molds as causative organisms as well, especially in treatment-resistant conditions. These molds are commonly found in African and Asian countries, as well as the Caribbean islands, Central America, South America, and parts of the United States [16]. *T. mentagrophytes* has been reported to be the most common cause of tinea infection in India, followed by *T. rubrum* and *T. interdigitale* [14]. While in North America and Europe, *T. rubrum* is the most common dermatophyte pathogen implicated in tinea, closely followed by *T. interdigitale* [17].

*T. rubrum* has been reported to be the main cause for chronic dermatophytosis infection [18]. A reason for this could be the uncontrolled use of antifungal medications, which can result in a selective pressure allowing a resistant strain to prevail within a population. In one study, *T. rubrum* was shown to develop resistance to fluconazole and itraconazole upon prolonged drug exposure. Analysis of minimum inhibitory concentration (MIC) values confirmed the inclination of *T. rubrum* to acquire resistance against fluconazole when compared with itraconazole. This study also reported patterns of cross-resistance between these two azole antifungals. The underlying mechanisms that can contribute to the development of *T. rubrum* resistance include increased drug efflux, decreased drug uptake, structural target site modifications, and the production of biofilms [19].

*T. tonsurans*, on the other hand, was initially native to Southeast Asia and Australia, but quickly expanded to the rest of the world through colonization, migration, and sports-related travel. *T. tonsurans* can live on household items and easily transmit infection through shared objects [20]. Interestingly, the prevalence of *T. tonsurans* is now increasing worldwide. In the United States, *T. tonsurans* is the primary cause of tinea capitis [21]. Additionally, in Germany, there is presently an increasing prevalence of tinea capitis caused by *T. tonsurans* and fellow anthropophilic pathogens *T. violaceum* and *T. soudanense* [22]. One possible explanation for this is the inadequate treatment of infections such as tinea capitis. For example, using griseofulvin, which is more effective at inhibiting *Microsporum* spp. than *Trichophyton* spp., in management of such cases may result in treatment failure, chronicity, and spread of infection [23].

## 3. Clinical Perspectives of Tinea

From a clinical perspective, dermatophyte infection, known as tinea, is further classified based on the anatomical region of the body affected (Table 1) [6]. Additionally, tinea infections can be transmitted from both humans and pets [24]. *T. tonsurans* and *Microsporum canis* are mainly known to cause tinea capitis infections [11]. Furthermore, tinea corporis (ringworm) is most commonly caused by *T. rubrum*, *T. mentagrophytes*, and *T. tonsurans* [25]. Tinea corporis can also be caused by contact with infected pets, though, the most common causative organism in this scenario is *M. canis* [24]. *T. rubrum* is the most common cause of tinea cruris (jock itch) around the globe, though *T. mentagrophytes* infections have been increasing in certain areas [26,27,28]. *Trichophyton* organisms have been found to affect male and female children equally. However, *M. canis* more commonly affects males [24].

Similarly, onychomycosis is commonly caused by dermatophytes (60–70% of the cases) and less commonly by other non-dermatophytes (mold and yeast). Several studies have shown that the majority of onychomycosis cases are caused by *T. rubrum*, followed by *T. mentagrophytes* [37,38]. Interestingly, in a study conducted in Iran that included 1284 microscopically positive onychomycosis cases, the main causative organism was *Candida albicans*. This was followed by *Trichophyton interdigital* and *Aspergillus flavus*. This may suggest a regional factor that can affect the prevalence of this type of infection [39].

The socioeconomic status of individual countries was reported to have an impact on the type of dermatophyte infection encountered in clinical practice. For example, tinea capitis is more prevalent in developing countries, while the prevalence of tinea pedis and onychomycosis is higher in developed countries [40]. Tinea pedis is a common fungal infection seen worldwide, with the most prevalent dermatophytes isolated in these cases being *T. rubrum*, *T. mentagrophytes*, and *Epidermophyton floccosum* [18,34,35,36]. This infection has been growing over recent years, yet the underlying pathogenesis is not definitively known [41,42]. However, tinea pedis was shown to be more prevalent in the adult-aged population, especially in males [34,35]. While tinea pedis and onychomycosis are prevalent around the globe, these infections are less common in India and rural Africa [36].

In the United States and the United Kingdom, *T. tonsurans* is the most common causative organism of tinea capitis infection [43,44]. Additionally, *T. tonsurans* can cause a type of tinea corporis infection known as tinea gladiatorum, which is common in athletes participating in direct contact sports, such as wrestlers. The average prevalence of tinea gladiatorum among wrestlers in the United States, Iran, and Turkey is 34.29% [20].

## 4. Standard Treatment of Tinea Infections and Current Limitations

The standard treatment of tinea infections is largely topical with azoles or allylamines. Tinea capitis and onychomycosis are more difficult to treat and typically require systemic oral therapy. Systemic therapy may also be utilized in chronic, refractory, or severe tinea infections (Table 2).

Medications that are commonly used to treat infections caused by *T. rubrum* include terbinafine, itraconazole, amorolfine, and ciclopirox [60]. Terbinafine, available as both a topical and oral medication, has long been a standard drug of choice for the treatment of tinea infections [61]. The topical form is available as terbinafine 1% cream and is used as the first line treatment for most tinea corporis, tinea cruris, and tinea pedis infections [6]. On the other hand, oral therapy is mainly used for more resistant conditions such as tinea capitis and onychomycosis, or for areas of extensive skin infection. This is especially true for patients who fail topical therapy or are immunocompromised [62]. In adults, oral terbinafine 250 mg once daily is the recommended first line treatment for onychomycosis. Itraconazole and fluconazole, available as oral medications, are other alternatives used as second line agents for conditions that require systemic treatment [6].

In a Cochrane review conducted in 2017, comparing a terbinafine treatment group to an azole treatment group, terbinafine was shown to be more effective at treating onychomycosis compared with azoles. Additionally, both groups had similar adverse reactions of headache, nausea, and viral infection [63]. For terbinafine, the side effects that are commonly observed include GI disturbance, headache, and taste alteration [64]. Hepatotoxicity, while rare, is a potentially life-threatening complication of both terbinafine and itraconazole [64,65]. Beside the above complications, drug–drug interactions may influence serum itraconazole levels and must be acknowledged before prescribing the medication [66].

The FDA has only approved oral terbinafine and itraconazole for the treatment of onychomycosis. However, fluconazole is also used as an off-label alternative treatment for onychomycosis. Pulse dosing regimens and booster therapy may also be utilized in the treatment approach, particularly with itraconazole. On the other hand, for topical treatments, only ciclopirox 8% nail lacquer, efinaconazole 10% solution, and tavaborole 5% solution have been approved by the FDA for management of onychomycosis [67].

## 5. Treatment Failure and Diagnostic Challenges

Numerous factors may contribute to treatment failure of SFIs including misdiagnosis, inappropriate use of antifungals, and the development of antifungal resistance. These conditions may be referred to as “recalcitrant dermatophytosis” [68]. Recurrent chronic dermatophytosis may result from intra-familial infection, prior history of inappropriate corticosteroid use, low treatment compliance, or premature treatment termination. Poor hygiene practices such as infrequent bathing, changing of undergarments, and washing of clothing, as well as sharing items like footwear, towels, and bedsheets, have also been noted as potential contributing factors to persistent infection. Dermatophytes can easily spread in the home environment, and it is important to recognize that asymptomatic carriers may transmit infection as well [14,69]. One study by Ghannoum et al. investigated the transmission of dermatophyte infection among infected households utilizing molecular typing and found that 44% of the investigated households had intra-familial transmission of infection [69].

Many dermatologic conditions may mimic tinea infections, thus, adequate diagnosis of dermatologic presentations is crucial for appropriate treatment, especially when considering systemic therapy [62]. For example, non-dermatophyte infections of the skin folds, such as cutaneous candidiasis, may mimic those of dermatophytes; however, they both have different treatments. Topical treatment with clotrimazole or miconazole for these infections is preferred over terbinafine [70,71]. Moreover, the actual dermatologic condition could be something else other than infection. This can be observed with some types of tineas, such as tinea corporis, which may present similarly to other conditions including atopic dermatitis, discoid eczema, annular psoriasis, pityriasis rosea, subacute cutaneous lupus erythematosus, and erythema annulare centrifugum [72,73]. In this scenario, tinea infections may be treated inappropriately with topical corticosteroids or immunosuppressive drugs. This event is referred to as tinea incognito [62]. Corticosteroids can initially suppress the inflammation of tinea infections; however, this is only temporary and most often leads to further inflammation and flare of disease [14].

In recurrent infections, it is important for providers to consider potential dermatophyte reservoirs elsewhere on the body and perform a full skin examination, as a single infection can easily be spread among one host. For example, onychomycosis can further spread to the foot, resulting in tinea pedis, while also spreading to the hand, trunk, and groin. Additionally, animals and household pets may also serve as dermatophyte hosts and should be considered in the treatment approach, especially when considering infection by *M. canis* [62].

Onychomycosis can also be confused with other conditions such as psoriasis, lichen planus, subungual melanoma, and bacterial infections [74]. Thus, generally, utilization of the available laboratory tests to properly identify dermatophyte infections is advisable.

## 6. Emergence of Drug Resistant Organisms

A factor that contributes to the observed increase in the incidence of dermatophyte infections is the development of antifungal resistance. Recently, there has been an increasing emergence of antifungal-resistant dermatophyte infections across the globe [75]. This resistance was initially noted in India but has also now been reported in parts of Europe [76]. Additionally, similar observations from other countries such as Iran, Japan, and China have been also reported [14,77,78], as well as recently in the United States [79,80]. The predominant causative dermatophyte for these infections has been reported to be *T. mentagrophytes genotype VIII*, recently designated *T. indotineae.*

**Figure 1:**
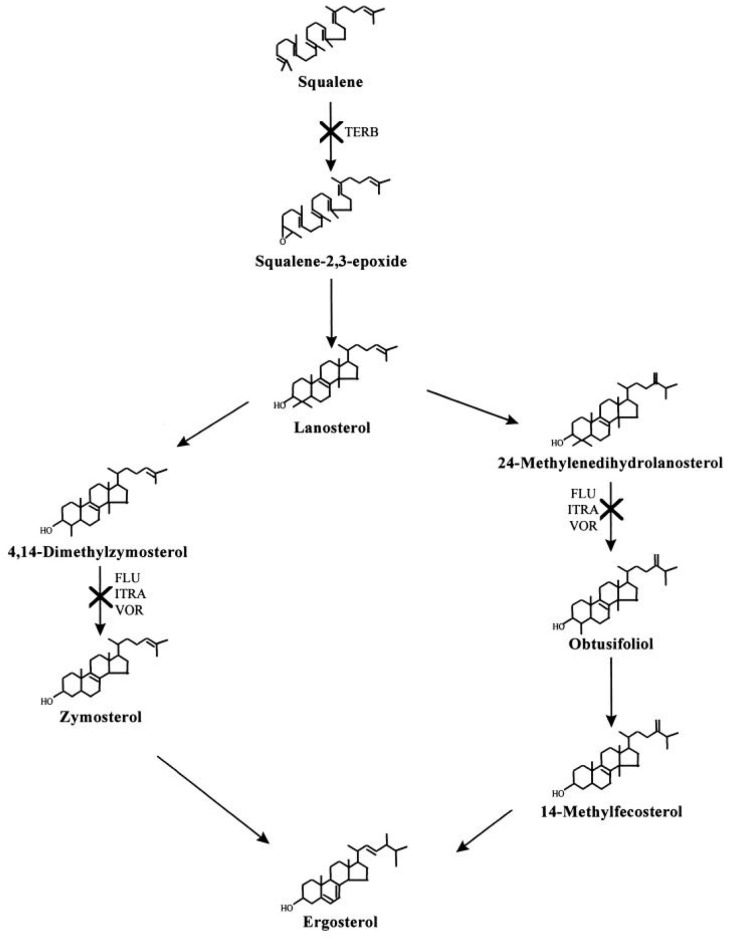
Ergosterol biosynthetic pathway: antifungal sites of action [83].

Resistance to terbinafine, one of the most utilized antifungals, has been reported throughout the literature over the past decade. Terbinafine, a first-line treatment for dermatophytosis, acts by inhibiting the enzyme squalene epoxidase. This enzyme is responsible for the synthesis of ergosterol, which is a necessary component of the fungal cell membrane (Figure 1). In refractory dermatophytosis, terbinafine resistance has been attributed to a point mutation in the squalene epoxidase gene [17]. The F397L and L393F point mutations have been detected in terbinafine-resistant *T. rubrum* and *T. mentagrophytes* strains [81]. Similarly, in Delhi, India, a case series evaluating tinea cruris and tinea corporis patients analyzed 20 *T. interdigitale* strains and reported elevated MIC values for terbinafine. All strains were also reported to have a squalene epoxidase point mutation at either F397L or L393F [75]. These mutations have additionally been reported in another study conducted in Denmark, in which isolates obtained from 14 cases demonstrated resistance to terbinafine. The *T. rubrum* and *T. interdigitale* isolates reported in this study also harbored additional squalene epoxidase point mutations such as L393S, F415S, H440Y F484Y, and I121M V237I [82].

The Ser395Pro (TCT → CCT) point mutation and amino acid substitution are also common in terbinafine-resistant dermatophytes [82]. In Lausanne, Switzerland over 2000 *Trichophyton* strains were evaluated for terbinafine resistance. One percent of these strains demonstrated decreased sensitivity to terbinafine. These isolates were found to carry squalene epoxidase point mutations with a single amino acid substitution at four locations: Leu393, Phe397, Phe415, and His440 [84]. However, these mutations have been found to occur most commonly at Leu393 and Phe397 [85]. These mutations have also been detected in *T. indotineae* isolates as reported by Singh et al. [86]. Additional mutations that have been associated with *T. indotineae* include Ala448Thr amino acid substitution in *erg1*. The two tested isolates in that study exhibited an intermittent drug response to terbinafine [87].

While terbinafine resistance is most notable, resistance to azole drugs is also prevalent. The primary mechanism behind azole resistance in dermatophytes is increasing drug efflux, though decreasing drug uptake and target site structural alterations have also been noted [88]. *T. rubrum* has been shown to demonstrate resistance against itraconazole and voriconazole due to the overexpression of two genes, *TruMDR2* and *TruMDR3*, which encode multidrug ABC transporters. *T. indotineae* has similarly shown resistance against itraconazole and voriconazole due to an overexpression of the *TinCYP51B* gene, which encodes sterol 14α-demethylase, an essential enzyme responsible for the conversion of lanosterol to a precursor of ergosterol [85] (Table 3).

## 7. The Impact of Increasing Trends of Fungal Infections and Growing Antifungal Resistance

The rising number of fungal infections and increasing antifungal resistance are becoming a major global health challenge and an economic burden. Every year, fungal infections result in over 1.5 million deaths worldwide. Mortality is higher in those who are immunocompromised, as these patients have an increased risk of developing invasive and deep dermatophyte infections due to reduced local cellular immune response [89]. These infections are characterized by extensive dermal and subcutaneous tissue invasion with potential spread to the lymph nodes [90,91]. Based on a systematic review that was published in 2021, 160 cases of invasive fungal infections have been identified in the years between 2000 and 2020 [92]. Interestingly, those with immune-related genetic deficiencies (such as *CARD9* or *STAT3*) are more prone to have invasive dermatophytosis at an age younger than 40 years old. Although the number of reported cases is not large, the growing number of immunocompromised patients presents concerns that call for special attention.

As for economic costs, fungal infections have become a significant global expense. In 2018, the United States spent approximately USD 6.7 billion in costs associated with fungal infections [89]. Fungal infections caused by dermatophytes alone account for at least USD 500 million in healthcare costs [11]. An important aspect likely contributing to the high costs associated with fungal infections is a lack of efficacious treatment. One study by Panackal et al. performed a cross-sectional analysis of ambulatory visits within the United States and reported that polyenes, while ineffective at treating tinea infections, were prescribed by physicians in significant amounts. This indicates a potential need for further provider education on the treatment of dermatophytosis [3].

Dermatophytosis has previously been associated with lower socioeconomic status. Racial differences have also been reported, with Black patients having a higher incidence of some tinea infections [3]. As infections continue to rise, the racial and economic disparities associated with dermatophytosis will only increase. Multiple studies have reported the disparity of tinea capitis disproportionately affecting Black adolescents [3,93,94]. One of these studies additionally reported that children in lower socioeconomic strata were also disproportionately affected by tinea capitis [3].

## 8. Management Prospective and Alternative Treatments

To address management of SFIs, there is a need for proper identification of the organism causing the infection. Furthermore, development of rapid laboratory assays that can detect the terbinafine resistant mutations can aid in drug selection and reduce the incidence of treatment failure.

Fungal culture, the traditional method used for identifying dermatophytes, has several limitations. It is a lengthy process that can take several weeks until the fungal culture results are available, with the potential for having a false negative result [2]. In addition, morphological interspecies differentiation can be difficult in some cases such as *T. indotineae*, *T. mentagrophytes*, and *T. interdigitale*, and in general, requires great experience for accurate identification [95,96]. Molecular diagnostic methods, on the other hand, are becoming a more preferable option, as they can provide quicker and more accurate results [97]. Although this may require special training and advanced equipment, the benefits of utilizing such techniques outweigh this hurdle. Several methods have been used in this regard including polymerase chain reaction (PCR) based on internal transcribed spacer (ITS) sequencing, real-time PCR, DNA microarray, and next-generation sequencing [98]. Currently, studies have shown that the best accuracy can be obtained by using both conventional and molecular techniques [97]. Thus, incorporating molecular techniques into routine diagnosis of dermatophytosis can help in overcoming the limitations of the traditional methods.

Several treatment options have been studied over the past years including newer azoles, such as efinaconazole, luliconazole, and tavaborole, a class of drugs known as oxaborole antifungals. Additionally, other alternative treatments, such as laser therapy, are currently being tested for the treatment of SFIs.

Efinaconazole is a triazole used in the treatment of onychomycosis as a topical 10% solution [56,57,58,59]. Conversely, luliconazole is an imidazole that is available as a 1% topical cream [99]. Efinaconazole has been shown to be effective in treating onychomycosis and may be more effective than other comparable antifungals due to its activity against a wide variety of superficial fungal pathogens [59,100]. It is applied once daily to the affected toenail(s) for 48 weeks. In vitro and in vivo studies have shown efinaconazole to be effective against *T. rubrum* and *T. mentagrophytes* isolates with MIC values of 0.06 μg/mL or less against ≥ 90% of the tested isolates [100].

Luliconazole has also demonstrated potent activity against dermatophytes. In a study by Wiederhold et al., luliconazole had a geometric mean MIC of 0.00022 μg/mL against 320 clinical isolates, compared with 0.0194 to 0.3107 μg/mL observed with amorolfine, ciclopirox, and terbinafine [101]. Furthermore, it demonstrated good activity in the treatment of dermatophytosis compared with terbinafine [102].

Tavaborole is another promising topical agent that can be used for the treatment of onychomycosis [103]. Tavaborole has shown activity in eliminating the fungal infection in clinical trials [104,105,106,107,108]. In two phase 3 clinical trials, tavaborole achieved a 6–9% complete cure rate [109]. Additionally, in a phase 4 trial, following 52 weeks of treatment, 14.9% of the patients achieved a complete/almost complete cure [108].

Alternative treatments for onychomycosis have included diode, erbium glass, carbon dioxide, and Neodymium-doped Yttrium Aluminum Garnet (Nd:YAG) laser treatments [110]. Laser therapy with a Nd:YAG 1064 nM laser may be a promising treatment modality in diabetic patients [111]. However, laser therapy overall has not been shown to be as effective as traditional topical or oral treatments [112]. While they have fungicidal effects, lasers have lower cure rates and require a long duration of treatment with multiple sessions. Additionally, laser therapy may be painful for some patients and is a costly financial investment. Ultimately, laser therapy is not recommended as a first line treatment [67]. Looking forward it is necessary to recognize the importance of both antifungal stewardship and susceptibility testing to improve patient outcomes and combat growing antifungal resistance.

## 9. Conclusions

It is evident that an increasing number of antifungal-resistant dermatophyte infections are posing a major global health and economic challenge, in combination with the growing number of non-dermatophyte fungal infections. Moving forward, these growing trends of resistance must be adequately addressed through innovative research with the development of new pharmacologic treatments or alternative therapies. In the clinical setting, it is important for providers to be aware of the various dermatophyte organisms that commonly cause tinea infections and to be aware of the strains that are becoming resistant to treatment. Adequate diagnosis and treatment of tinea infections will be a critical factor in reducing the number of antifungal-resistant dermatophyte strains.

## Figures and Tables

**Table 1:** Clinical classification of tinea infections [29].

Tinea Infection	Body Area Affected	Most Common Causative Pathogens
Tinea Capitis	Head and scalp	*T. tonsurans*, *Microsporum canis* [11]
Tinea Corporis	Trunk and extremities	*T. rubrum*, *T. mentagrophytes*, *T. tonsurans* [25]
Tinea Cruris	Groin, pubic region, intertriginous anogenital region	*T. rubrum*, *T. mentagrophytes* [26,27,28]
Tinea Faciei	Face	*T. rubrum*, *T. mentagrophytes* [30,31]
Tinea Barbae	Beard and mustache area	*T. verrucosum*, *T. rubrum*, *T. mentagrophytes* [32]
Tinea Manuum	Hands	*T. rubrum* [33]
Tinea Pedis	Feet	*T. rubrum*, *T. mentagrophytes*, *Epidermophyton floccosum* [18,34,35,36]
Onychomycosis (Tinea Unguium)	Nails	*T. rubrum*, *T. mentagrophytes* [37,38]

**Table 2:** Clinical viewpoints: systemic and local therapies for tinea infections.

Tinea Infection	Systemic Therapy	Local Therapy
Tinea Capitis [24]	Terbinafine or Griseofulvin. If kerion is present, add steroids.	Not recommended. Itraconazole or Fluconazole may be used in some cases.
Tinea Corporis [45,46]	Indicated for severe infection caused by *T. rubrum*. Terbinafine, Itraconazole, Fluconazole, or Griseofulvin. Terbinafine is indicated for Majocchi Granuloma.	Azoles or Allylamines
Tinea Cruris [27,47]	Indicated for chronic or recurrent infection. Terbinafine, Itraconazole, or Fluconazole.	Azoles or Allylamines
Tinea Faciei [30,48]	Indicated for severe or refractory infection or involvement of vellus hair	Azoles or Allylamines
Tinea Barbae [32]	Terbinafine, Itraconazole, Fluconazole, or Ketoconazole	Azoles or Allylamines as adjunct therapy
Tinea Manuum [33,49,50]	Indicated for co-infection of the nail, two feet-one hand syndrome, and chronic or recurrent infection. Terbinafine or Itraconazole may be effective.	Azoles or Allylamines
Tinea Pedis [6,50,51,52,53]	Indicated for treatment-resistant infection. Terbinafine, Itraconazole, Fluconazole, Ketoconazole, or Griseofulvin.	Indicated for uncomplicated or mild interdigital infection. Azoles or Allylamines. Luliconazole or Naftifine may be used for interdigital infection. Initial treatment with topical corticosteroids may be beneficial.
Onychomycosis (Tinea Unguium) [54,55,56,57,58,59]	Indicated for moderate to severe infection. Terbinafine or Itraconazole. Avoid Griseofulvin (lower efficacy) and Ketoconazole (hepatotoxicity).	Indicated for mild to moderate infection. Efinaconazole, Ciclopirox, or Amorolfine.

**Table 3:** Resistance mechanisms of antifungal-resistant dermatophytes.

Dermatophyte Pathogen	Resistance Mechanisms	Primary Associated Antifungal(s)
*T. indotineae*	F397L, L393F, F415S, or H440Y squalene epoxidase gene point mutations [84,86], Ala448Thr amino acid substitution in *erg1* [87], overexpression of *TinCYP51B* gene [85]	Terbinafine, Itraconazole, Voriconazole
*T. interdigitale*	F397L, L393F, F415S, H440Y F484Y or I121M V237I squalene epoxidase gene point mutations [81]	Terbinafine
*T. mentagrophytes*	F397L or L393F squalene epoxidase gene point mutations [81]	Terbinafine
*T. rubrum*	F397L, L393F, F415S, H440Y F484Y or I121M V237I squalene epoxidase gene point mutations [81], azole efflux pump (i.e., overexpression of *TruMDR2* and *TruMDR3* genes) [85]	Terbinafine, Itraconazole, Voriconazole

## Data Availability

Not applicable.
